# Repurposing Saquinavir for Host-Directed Therapy to Control Mycobacterium Tuberculosis Infection

**DOI:** 10.3389/fimmu.2021.647728

**Published:** 2021-03-26

**Authors:** David Pires, Sofia Valente, Marta Calado, Manoj Mandal, José Miguel Azevedo-Pereira, Elsa Anes

**Affiliations:** Host-Pathogen Interactions Unit, Research Institute for Medicines, iMed-ULisboa, Faculty of Pharmacy, Universidade de Lisboa, Lisboa, Portugal

**Keywords:** saquinavir, protease inhibitors, tuberculosis, HIV-co-infection, host directed therapies

## Abstract

Despite the available antibiotics, tuberculosis (TB) has made its return since the 90’s of the last century as a global threat mostly due to co-infection with HIV, to the emergence of drug resistant strains and the lack of an effective vaccine. Host-directed strategies could be exploited to improve treatment efficacy, contain drug-resistant strains, improve immune responses and reduce disease severity. Macrophages in the lungs are often found infected with *Mycobacterium tuberculosis* (Mtb) and/or with HIV. The long-term survival of lung macrophages infected with Mtb or with HIV, together with their ability to produce viral particles, especially during TB, makes these niches major contributors to the pathogenicity of the infection. Among the available drugs to control HIV infection, protease inhibitors (PIs), acting at post-integrational stages of virus replication cycle, are the only drugs able to interfere with virus production and release from macrophages during chronic infection. For Mtb we recently found that the pathogen induces a general down-regulation of lysosomal proteases, helping bacteria to establish an intracellular niche in macrophages. Here we found that the PI saquinavir, contrary to ritonavir, is able to induce an increase of endolysosomal proteases activity especially of cathepsin S in Mtb infected macrophages and during co-infection with HIV. Our results indicate that saquinavir treatment of infected macrophages led not only to a significant intracellular killing of Mtb but also: (i) to an improved expression of the HLA class II antigen presentation machinery at the cell surface; (ii) to increased T-lymphocyte priming and proliferation; and (iii) to increased secretion of IFN-γ. All together the results indicate saquinavir as a potential host directed therapy for tuberculosis.

## Introduction

*Mycobacterium tuberculosis* (Mtb) the causative agent of tuberculosis (TB) and the AIDS-associated human immunodeficiency virus (HIV), have in common macrophages (Mø) as immune cell reservoir. Both pathogens alter the Mø microbicidal and immune-activating functions and convert these cells into intracellular survival niches (1). In the case of Mtb, the WHO estimates that one quarter of the human population is latently infected and among these, 10% will develop the active disease. From the latently infected group about 600,000 people are estimated to be carriers of multidrug-resistant (MDR) and extensively drug-resistant Mtb strains (XDR) (2). Main contributors to TB activation from latency are immunosuppressive conditions, especially HIV co-infection, malnutrition and aging.

While for HIV the infection became a chronic controlled situation with the available medicines, for TB and particularly during co-infection with HIV the scenario is a global threat for humankind. This includes, as aforementioned, (A) the increased MDR and XDR strains to current available antibiotics; (B) the condition that Mtb exacerbates HIV infection and vice versa leading to TB activation from latency; and (C) the fact that co-infected individuals contribute to viral spread and to MDR and XDR strains transmission (2–4). All together this led us to search for host targets that may be manipulated during infection to boost the immune responses blocked by the pathogens as an alternative therapeutic strategy to current antimicrobials. In this context, the repositioning of drugs represents a useful strategy in the search for new fast therapeutic approaches for TB control. Furthermore host-directed strategies could be exploited to improve treatment efficacy and outcome and reduce disease severity and mortality.

Combined antiretroviral therapies have been applied in HIV-infected patients for more than two decades and include a cocktail of nucleoside reverse transcriptase inhibitors (NRTIs), non-NRTIs (nNRT1s), protease inhibitors (PIs), and integrase inhibitors (5). These were shown to efficiently suppress HIV replication, leading to partial immune restoration and turning AIDS into a chronic infection. A threat to this controlled situation arises from the fact that HIV in addition to infect CD4^+^ T-lymphocytes also infects Mø. While the absolute number of infected Mø in the body is relatively low compared to CD4^+^ T-cells this is not the case for HIV infected Mø in the lungs (6) particularly during co-infection with Mtb (7). Furthermore alveolar Mø simultaneously infected with HIV and Mtb, were isolated from a patient co-infected with both pathogens (8). The long-term survival of lung Mø infected with these pathogens, together with their ability to exacerbate the infection by each other, turns these viral reservoirs into a challenge to HIV eradication since they continue producing virus in this tissue despite antiretroviral therapy (3, 4). Among the available drugs to control HIV infection, protease inhibitors (PIs), acting at post-integrational stages of virus replication cycle (9), are the only drugs able to interfere with virus production and release from Mø during chronic infection (10). The anti-viral activity of PIs is based on inhibition of the HIV aspartic protease, responsible for the cleavage of the Gag/Pol polypeptide and the structural viral core proteins leading to the production of immature viral particles, the inhibition of viral replication and cell-to-cell spreading (10–12).

PI were recently shown to directly act as modulators of endolysosomal proteases activity, namely of cysteine cathepsins in human CD4^+^ T-cells and in antigen presenting cells (APCs) as dendritic cells and Mø (13). Curiously, while saquinavir (SQV) activates omni-cathepsins enzymatic activity (omnicathepsins includes cathepsins B, L and S), ritonavir (RTV) displayed the opposite effect on cells obtained from non-infected individuals.

For Mtb we previously demonstrated that during infection of human Mø, a general down-regulation of cathepsins gene expression, concomitant with a decreased protease activity, occurs either in resting M0 or in IFN-γ M1 activated cells (14, 15). This may be a strategy used by the pathogen to manipulate the host microbicidal responses in order to survive intracellularly in these immune cells and to prevent antigen presentation. Here we found that SQV, contrary to RTV, is able to enhance the omnicathepsins protease activity including a very significant increase in cathepsin S activity in Mtb infected Mø. The enhancement of the catalytic activity was able to overcome the enzymatic inhibition induced by the pathogen in a three-fold magnitude. The same was observed during HIV co-infection. Our results indicate that SQV treatment during Mtb infection led not only to an exacerbated intracellular killing of the bacteria but also to an improved expression of the HLA class II antigen presentation machinery at the cell surface, to CD4^+^ T-lymphocyte priming and proliferation as well as to increased secretion of IFN-γ. All together the results indicate SQV as a potential host directed therapy for tuberculosis.

## Materials and Methods

### Cells and Culture Conditions

Human monocyte-derived Mø were obtained by isolating CD14^+^ monocytes from buffy coats of healthy blood donors provided by the national blood institute (Instituto Português do Sangue e da Transplantação, Lisbon, Portugal) following a protocol established between Dr. Anes (Faculty of Pharmacy, University of Lisbon) and the Portuguese Institute for Blood, allowing access to buffy coats from healthy blood donors, for scientific research and academic purposes. The supplier provided no personal details from the donors. The cells were isolated using the MACS cell separation system (Miltenyi Biotec). Briefly, the mononuclear cell fraction was isolated using Ficoll-Paque PLUS (GE Healthcare) density gradient medium. This fraction was incubated with anti-CD14 magnetic beads and then passed across the MACS magnetic columns for positive monocyte selection. To induce differentiation to Mø, the isolated monocytes were allowed to adhere to 48- or 96-well plates at 1.5 × 10^5^ or 5 × 10^4^ cells per well, respectively for 2 h at 37°C, 5% CO_2_, in RPMI-1640 medium (HyClone, GE Healthcare). Following adherence, the medium was supplemented to achieve a final concentration of 10% (v/v) FBS (HyClone, GE Healthcare), 1 mM sodium pyruvate (HyClone, GE Healthcare), 10 mM HEPES (HyClone, GE Healthcare), 0.1% β-mercaptoethanol (Gibco), and 20 ng/mL recombinant human M-CSF (Biolegend). Differentiation lasted for 7 days and medium was renewed every three to four days until day 7. Purity of the isolated culture was verified by flow cytometry.

### Bacterial Cultures and HIV Isolates

*Mycobacterium tuberculosis* H37Rv (ATCC 27294), H37Rv GFP-expressing strain and *Mycobacterium bovis* BCG Pasteur (ATCC 35734) were grown in Middlebrook’s 7H9 medium supplemented with 10% OADC enrichment (Difco), 0.02% glycerol and 0.05% tyloxapol at 37°C (15). Preceding the infections, bacterial cultures on exponential grown phase were centrifuged and washed in phosphate-buffered saline (PBS). Bacteria were resuspended in RPMI-1640 medium (Mø culture medium) without antibiotics. In order to dismantle bacterial clumps, the bacterial suspension was treated by ultrasonic bath for 5 min. Residual clumps were removed by 1-minute centrifugation at 500 × *g*. Single-cell suspension was verified by fluorescence microscopy and quantified by optical density at 600 nm.

Primary HIV-1 isolate UCFL1032 was obtained after cocultivation of infected patient’s peripheral blood mononuclear cells (PBMCs) with PHA-stimulated PBMCs from uninfected individuals. Viral stocks were established in PBMCs from low-passaged supernatants of original cultures, aliquoted and maintained at -80° C until used. Viral concentration was measured by reverse transcriptase (RT) activity using an enzyme linked immunosorbent assay (Lenti-RT kit, Caviditech, Uppsala, Sweden). HIV-1_UCFL1032_ was characterized both genetically and phenotypically: it belongs to subtype B and uses CXCR4 coreceptor to enter host cells. It has the ability to enter Mø that produce low amounts of viral progeny upon infection, a phenotype similar to what is described during the course of patients Mø infection (16). This isolate is part of viral library created and maintained in our laboratory since the late Eighties, where a significant amount of HIV-1 and HIV-2 were characterized (17).

All experimental procedures using live Mtb and HIV were performed in the Biosafety Level 3 laboratory at the Faculty of Pharmacy of the University of Lisbon, respecting the national and European academic containment level 3, laboratory management and biosecurity standards, based on applicable EU Directives. All procedures have been approved by the faculty’s biological safety committee.

### Treatment and Infection of Mø

Prior to infection, Mø were treated for 1 h with selected concentrations of saquinavir (SQV) (Merck Life Science) or ritonavir (RTV) (Merck Life Science) previously reconstituted in DMSO. Following pretreatment, the bacterial/viral suspension was added without removing the inhibitors. Mø were infected with a MOI of 1 of bacteria and inoculated with the equivalent of 1 ng of RT of HIV-1_UCFL1063_. After 3 h of infection at 37°C, 5% CO_2_, the cells were washed with PBS to remove free bacteria/virus and cultivated in fresh complete medium supplemented with SQV or RTV. The controls were treated with the same concentration of DMSO as carried during treatments.

Phagocytosis of the bacteria was evaluated by flow cytometry using *M. tuberculosis* H37Rv GFP-expressing strain and following the procedures below. Monitorization of HIV infection was performed by fluorescence microscopy. Macrophages were fixed with 4% paraformaldehyde 4% sucrose solution in PBS for 1 h and quenched by incubating with 50 mM NH_4_Cl in PBS. Cells were permeabilized with 0.1% Triton X-100 for 5 min and blocked with 1% BSA in PBS for 30 min. Cells were stained with anti-Gag antibody 1:100 (KC57, Beckman Coulter) in PBS BSA 1% for 1 hour, washed and then incubated with Alexa Fluor 555 Goat anti-Mouse IgG secondary antibody 1:1000 (Cell Signaling Technology) for 30 minutes. Coverslips were mounted using ProLong Gold Antifade Mountant (ThermoFisher Scientific) and visualized on a Zeiss Axioskop 40 fluorescence microscope. Analysis was performed on ImageJ software **(** **Supplemental Figure 1** **)**. To further confirm that the cell culture was infected with HIV, integration of the viral DNA into host genome was confirmed using nested polymerase chain reaction (PCR) as described (18). Briefly, a first round of PCR amplification was done using an *Alu*-specific sense primer in combination with a *gag* antisense HIV-1 specific primer; the PCR products were then subjected to a second amplification reaction targeting the HIV-1 R/U5 region of LTR, leading to an amplicon with 391 bp **(** **Supplemental Figure 1** **)**.

### Macrophage Viability

Macrophages seeded in 96-well plates were treated with SQV, RTV or DMSO for 3 days. Next, samples were washed and incubated with PrestoBlue (Invitrogen) resazurin-based solution at 37°C, 5% CO_2_, according to the manufacturer’s instructions. After 3 h of incubation, fluorescence emission was analyzed in a Tecan M200 spectrofluorometer. Non-treated cells were used as reference and cells treated with RTV 100 µg/mL were used as control for cell death.

### Enzymatic Activity of Cathepsins

Following 24 h of treatment and infection with *M. tuberculosis* H37Rv, or co-infection with HIV, Mø in a 96-well plate were washed with PBS and incubated in PBS with omnicathepsin (Z-FR-AMC, Z-Phe-Arg-AMC) (Enzo Life Sciences) or cathepsin S (Z-VVR-AFC) (BioVision) fluorogenic substrate for 1,5 h at 37°C in a Tecan M200 spectrofluorometer. Fluorescence readings were performed every 5 min. Essay specificity was verified by treating the cell lysates with general protease inhibitor E-64d or with specific cathepsin S inhibitor, provided in the kit.

### Bacteria Intracellular Survival

When required, infected cells in 96-well plates were lysed in 0.05% Igepal solution for 15 min. Serial dilutions of the resulting bacterial suspension were plated in Middlebrook 7H10 with 10% OADC (Difco) and incubated for 2-3 weeks at 37 °C before colonies were observable and counted under the microscope.

### Bacteria Growth Curves in Broth Medium

*M. tuberculosis* H37Rv in single-cell suspension were incubated in bacteria culture medium with selected concentrations of SQV, RTV and DMSO at 37°C, 5% CO_2_ for 15 days. The optical density at 600 nm was measured at discrete time points. Isoniazid (INH) was used as control for inhibition of growth.

### Flow Cytometry

Following 24 h of treatment and infection with *M. tuberculosis* H37Rv, Mø in 48-well plates were recovered with HyQTase cell detachment solution (HyClone, GE Healthcare). For the identification of apoptotic and necrotic cells Annexin V Apoptosis Detection Kit with PI (Cat # 640914, Biolegend) was used following the manufacturer’s instructions. Cells were incubated with annexin V and propidium iodide for 20 minutes, washed with the appropriate kit buffer and fixed in 4% paraformaldehyde for 1 h. Following fixation, cells were washed again in buffer and analyzed. For surface staining of HLA molecules, detached cells were promptly fixated for 1 h. Following fixation, cells were washed and incubated with Human TruStain FcX Fc receptor blocking solution (Biolegend) for 10 minutes and then stained for 20 min with antibodies specific for human HLA class I (Cat # 311422, Biolegend) and HLA class II (Cat # 361716, Biolegend) molecules. Samples were analyzed in Guava easyCyte™ 5HT flow cytometer.

### CD4^+^ T-Lymphocytes Proliferation

Autologous CD4**^+^** T-lymphocytes were obtained from the same healthy PPD^+^ donors according to the isolation protocol described above. Positive selection of the CD4**^+^** lymphocytes was performed using anti-CD4 magnetic beads (Miltenyi Biotec). Isolated lymphocytes were cultivated in 75 cm^2^ flask at 2 × 10^6^ cells per mL in RPMI-1640 medium (HyClone, GE Healthcare) supplemented with 15% (v/v) FBS (HyClone, GE Heaclthcare), 1 mM sodium pyruvate (HyClone, GE Heaclthcare), 10 mM HEPES (HyClone, GE Heaclthcare) and 20 UI/ml of human recombinant Interleukin 2 (Biolegend) for 3 days prior to the experiment. Immediately before the experiment the lymphocytes were stained with Carboxyfluorescein diacetate succinimidyl ester (Cat # 423801, Biolegend) following the manufacturer’s instructions. Macrophages infected with *M. tuberculosis* H37Rv or *M. bovis* BCG and treated for 24 h were washed and cocultivated with the lymphocytes at a ratio of 5 lymphocytes per macrophage for 5 days. CD4**^+^** lymphocytes were recovered after 5 days of coculture and analyzed using Guava easyCyte™ 5HT flow cytometer.

### IFN-γ Quantification

Supernatants from the previous assays were recovered following 24 h of infection and treatment and following an additional 5 days of coculture with CD4**^+^** lymphocytes and stored at -80 °C for posterior analysis of interferon-γ (IFN-γ) secretion. The quantification was performed by Sandwich Enzyme-Linked Immunosorbent Assay using ELISA Max Deluxe Set Human for IFN-γ (Cat # 430104, Biolegend) kits and following the manufacturer’s instructions. Absorbance was measured by Tecan M200 spectrofluorometer at 570 nm.

### Statistical Analysis

Data are presented as mean ± standard error except if stated otherwise. Statistical analysis was performed using SigmaPlot 12. Multiple group comparisons were made using ANOVA one parameter tests followed by pairwise comparisons of the groups using Holm-Sidak test. Two group comparisons were made using Student’s t-test. All the prerequisites of the tests were verified. The considered nominal alpha criterion level was 0.05 below which differences between samples were deemed significant.

## Results

### Treatment With Saquinavir Impacts Cysteine Cathepsins Enzymatic Activity in Mø Infected With Mtb and HIV

Protease Inhibitors (PI) prescribed to HIV-infected patients were previously found to directly manipulate the proteolytic activity of endolysosomal cysteine cathepsins in APCs isolated from healthy non-infected donors (13). Here in the context of infected Mø with Mtb or during co-infection with HIV, we first aimed to assess the effect of HIV PIs saquinavir (SQV) and ritonavir (RTV) on omnicathepsin proteolytic activities (which measure the combined activities of cathepsin B, L, and S). Cathepsins B, L and S are all involved in intracellular killing of pathogens internalized by Mø through phagocytosis/endocytosis (14). Cathepsin S, in addition, is strongly expressed in APCs and also operates in the endocytic pathway with proteolytic activities required for antigen and MHC class II processing (15, 19, 20).

The selected concentrations of SQV and RTV ranging from 5 to 20 μg/mL were based in previous studies (5, 13) concerning the average levels found in the plasma of people treated with a single daily dose of 5 to 10 μg/mL (13, 21–23). Here, Mø were treated with SQV or RTV one hour before infection (as detailed in methods) and the drugs were left in contact with cells during the whole assay. The cleavage of a peptidase-specific fluorogenic peptide substrate was measured over 1.5 hours, 24 h post-infection. Cells treated with omnicathepsin inhibitor E-64d were used as control.

**Figure 1:**
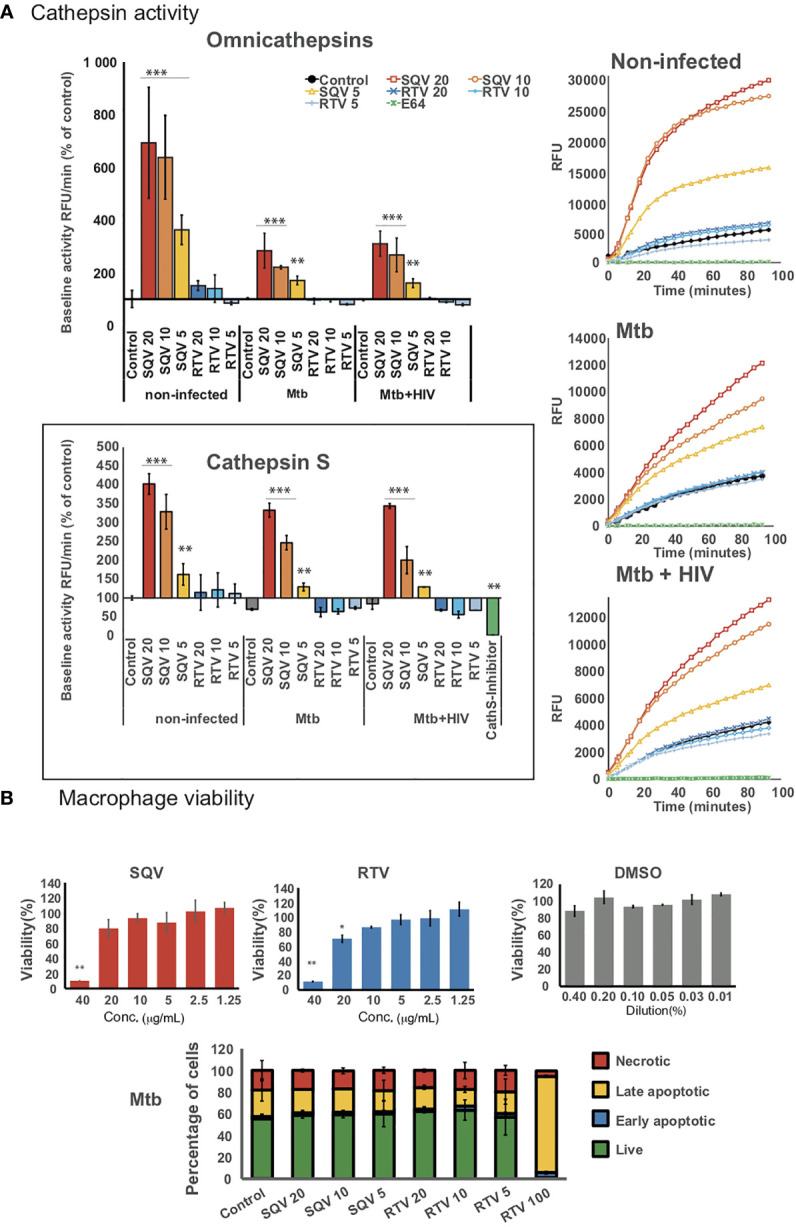
HIV PIs alter cathepsins’ activity in human macrophages infected with Mtb. **(A)** Omnicathepsin activity or cathepsin S activity alone were monitored with a specific fluorogenic substrate every 5 min in live cells pretreated with DMSO, RTV, SQV, or with specific inhibitors (E-64d or ZFL-COCHOO for cathepsin S). The slope of fluorescence emission in the presence of DMSO was represented as 100%, and the effect of each PI was calculated as a percentage of the DMSO control. Data are represented as average from three independent experiments and donors and data dispersion represented by the error bars as standard error (*P < 0.05, **P < 0.01, ***P < 0.001 relatively to control). **(B)** Cell viability (upper bar-plots) was measured in non-infected cells treated for 3 days with the PIs and using PrestoBlue resazurin-based solution by quantifying the emission of fluorescence in a plate reader. Cell death (lower bar-plot) was measured by flow cytometry after 24 h of infection using FITC-Annexin V and propidium iodide. Values show the average of three biological replicates from one representative experiment performed in triplicate while error bars depict standard deviation (*P < 0.05, **P < 0.001 relatively to control).

**Figure 1A** (upper panels) shows the effects of PIs on omnicathepsin proteolytic activities in Mø infected with Mtb or co-infected with HIV relatively to non-treated infected cells. Treatment with SQV led to a very significant increase of the proteolytic activity in a dose-dependent manner, while no effects were observed for RTV in all conditions tested. In non-infected cells **(** **Figure 1A** upper panels left and right) the effects of SQV on cathepsin kinetics was more exacerbated than in infected ones reinforcing our previous results that Mtb infection results in an overall decrease of cathepsins activity (14, 15). The kinetics when using the concentration of 10 μg/mL was near the saturation level by the end of 60 min treatment (**Figure 1A** upper right panel).

In parallel, we assessed the PIs effect on kinetics of cathepsin S activity alone using a cathepsin S cleavage-specific fluorogenic peptide substrate. As depicted in **Figure 1A** (lower panel) SQV strongly enhanced the proteolytic activity of cathepsin S in a dose dependent manner during Mtb infection and during co-infection with HIV contrary to RTV that presented kinetics similar to the control.

To confirm the effect of SQV and RTV in cell viability/cytotoxicity we performed the resazurin assay (**Figure 1B** upper panel) with results indicating cytotoxic effects on Mø viability at a concentration of 40 and 20 μg/mL for SQV and RTV, respectively, but without effects when using therapeutic concentrations of 5- 10 μg/mL. To further evaluate the impact on programmed cell death in infected Mø we used annexin-V and propidium iodide staining as markers for apoptosis and necrosis. We detected no increased toxicity on infected cells treated with concentrations of PIs ranging from 5 to 20 μg/mL, relatively to the control **(** **Figure 1B** lower panel; **Supplemental Figure 2** **)**.

### Treatment With HIV PI Saquinavir Results in Increased Mtb Killing in Primary Human Mø During Mono-Infection and During HIV Co-Infection.

Once established that PI treatment of infected cells did not impact apoptosis neither necrosis in the experimental conditions used and, therefore, would not interfere with the amount of live bacteria recovered from infected Mø, we next tested the effects of SQV and RTV on Mtb intracellular killing. Our hypothesis is that SQV strongly increasing the proteolytic activity of omnicathepsins may reverse and largely compensate the induced inhibitory effect observed during Mtb infection (14).

**Figure 2:**
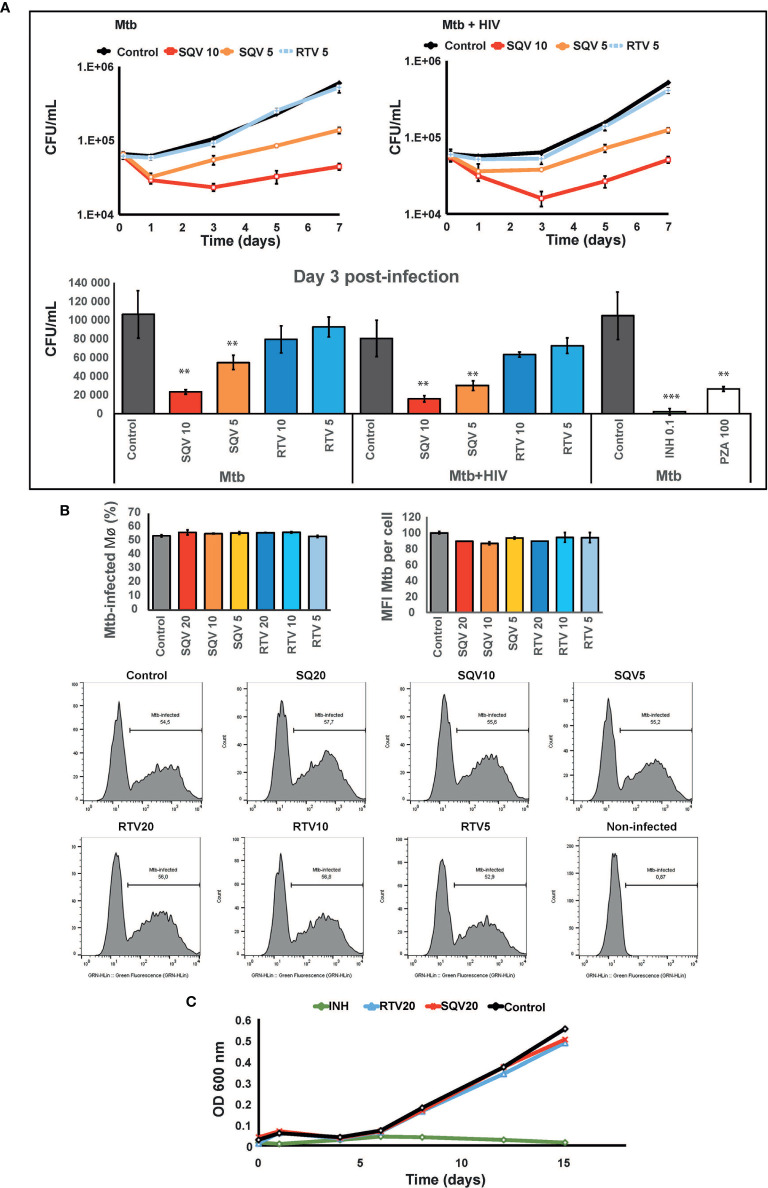
SQV decreases the intracellular survival of *Mycobacterium tuberculosis* (Mtb). **(A)** Intracellular survival of Mtb during mono- or co-infection with HIV along 7 days of infection. Data represents colony forming units (CFU) of intracellular bacteria recovered from Mø treated with the PI or DMSO control. Culture medium was changed on day 3 p.i. without addition of fresh PIs. Values depict mean CFU representative of three biological replicates from one representative experiment performed in triplicate. Error bars depict the standard deviation (P** < 0.01; ***P < 0.001 relatively to control). **(B)** Percentage of Mø infected with Mtb and median fluorescence intensity of Mtb per Mø were measured by flow cytometry in Mø pre-treated with the PIs and after 3 h of infection with GFP-expressing Mtb. Bar-plots depict the average of three biological replicates and the error bars depict the standard error. Raw values from one representative replicate are presented in the fluorescence intensity histograms. **(C)** Mtb growth curves in broth medium treated with PIs and incubated for 15 days. Values represent the optical density measured at discrete time points from one representative experiment performed twice. Isoniazid (INH) was used as control for inhibition of growth.

As shown in **Figure 2A** (upper and lower panel) pre-treatment with SQV in therapeutic concentrations significantly enhances the intracellular killing of Mtb during mono-infection or during HIV co-infection, in a dose-dependent manner (*P* < 0.001). No effects were observed using therapeutic concentrations of RTV. Since cells were pre-treated before infection and the PIs were added again just once after bacteria internalization into Mø, the impact on bacteria killing was mainly observed during the first 3 days of infection with a concomitant recover of the intracellular replication afterwards. The effects on intracellular killing of Mtb were similar to those achieved using pyrazinamide (PZA) at a minimal inhibitory concentration estimated *in vitro* of 100 μg/mL (**Figure 2A**, lower panel).

To confirm that PI treatment did not impact on the ability of Mø to internalize bacteria, we assessed Mø containing GFP expressing bacteria by flow cytometry (**Figure 2B**, **Supplemental Figure 2**). The results show that approximately 50% of Mø were infected (left panel) and the infected population was loaded with similar amounts of bacilli (right panel), independent of the concentration of PIs used.

Finally in order to disregard a microbicidal effect of the PIs directly in Mtb we evaluated the effect of higher concentrations of SQV and RTV than those used in *ex vivo* assays directly on bacteria replication in liquid media. Using turbidimetry assays at an OD of 600 nm, the *in vitro* growth of Mtb was similar to samples treated with 20 μg/mL either with SQV or RTV (**Figure 2C**). Isoniazid at a concentration of 0.1 μg/mL was used as control.

Altogether our results suggest that the Mtb intracellular killing effects of SQV are not attributed to a direct bactericidal effect of the drug but rather to an improved activity of omnicathepsins in the endocytic pathway. This is in accordance with our previous published results indicating that the limited non-toxic treatment with the omnicathepsin inhibitor E-64d helped Mtb survival in a 3 fold magnitude (14, 15).

### Treatment With HIV PI Saquinavir Results in Increased Surface Expression of HLA Class II Antigen Presentation Machinery and CD4^+^ T-Lymphocyte Proliferation

Appropriate innate immune responses lead to destruction of pathogens during phagocytosis but also to adaptive immune responses that are crucial to control infections. SQV was demonstrated to enhance cathepsin S activity in non-infected cells (13) and here we show that SQV significantly enhances cathepsin S activity in infected cells. Since SQV regulates the activity of cathepsin S it may also be implicated in endosomal antigen and HLA class II processing required for appropriate antigen presentation (19, 20). Previously we hypothesized that the noticeable Mtb-induced decrease in cathepsin S expression during infection might be linked to poor antigen processing and presentation, compromising the adaptive immunity response to infection (15). Here we further analyzed if SQV or RTV interfere with HLA class II antigen presentation machinery during infection, thus helping to improve the adaptive immune responses.

**Figure 3:**
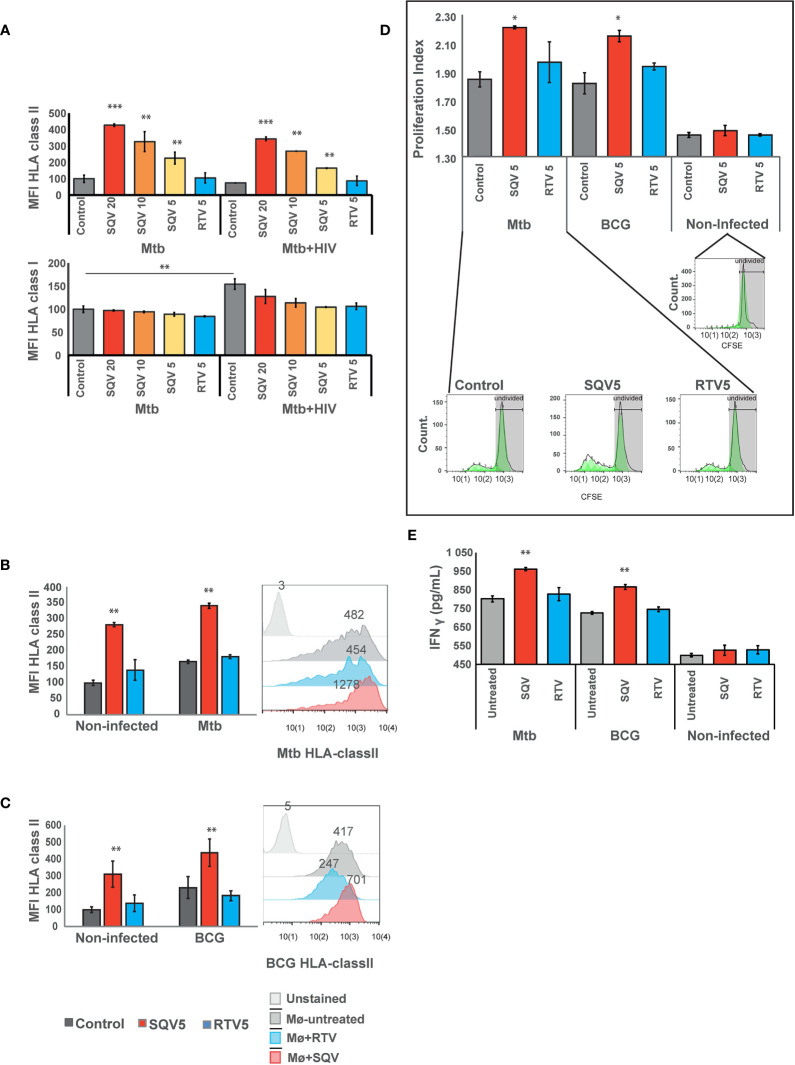
SQV results in increased expression of HLA class II antigen presentation machinery **(A)** Surface expression of human leukocyte antigen (HLA)-class II or class I on Mø infected with Mtb or co-infected with HIV. **(B)** Surface expression of human leukocyte antigen (HLA)-class II on Mø infected with Mtb or **(C)** BCG compared to non-infected Mø. HLAs were measured by flow cytometry after 24 h of infection. Values in bar plots represent the average of median fluorescence intensity measured on three biological replicates from one representative experiment performed in triplicate relative to control. Error bars depict standard deviation (**P < 0.001, relatively to control). Raw values from one representative replicate are presented in the fluorescence intensity histograms. **(D)** CD4**^+^** T-cell proliferation after 5 days of coculture with Mtb or BCG-infected Mø. Following 24 h of the infection, CFSE stained CD4**^+^** T-cells were added to the infected Mø culture. After 5 days of coculture, CD4**^+^** T-cells CFSE fluorescence was measured by flow cytometry. Values in bar plots represent the proliferation index (average number of divisions per cell) of CD4**^+^** T-cell (*P < 0.01, relatively to control). Histograms from one representative replicate of the different treatments infected with Mtb are presented in the bottom. The green areas represent the CD4**^+^** T-cell populations after each division as modeled by the software. **(E)** IFN-γ was quantified in the supernatant after 5 days of cocultures of Mø with CD4^+^T-lymphocytes by ELISA. Values depict mean concentration of three biological replicates from one representative experiment performed in duplicate. Error bars depict the standard deviation (**P < 0.01; ***P < 0.001, relatively to control).

For this, we first evaluated the effects of PIs during Mtb infection or during HIV co-infection and analyzed changes in the surface expression of HLA class II molecules compared to non PI-treated infected cells. For all concentrations tested, SQV treatment led to a significant increase of HLA class II expression at the cell surface as measured by flow cytometry (**Figure 3A** upper panel, **Supplemental Figure 3** **).** RTV used at the maximum therapeutic concentration found in plasma of treated patients achieved after a single dose administration (5 μg/mL), did not show any changes relatively to the control. In contrast, the lowest concentration of SQV (5 μg/mL) induced a significant increase of HLA class II presentation of endogenous antigens in non-infected cells, as observed in **Figure 3B**.

Cathepsin S, was demonstrated to be implicated in partial antigen processing for cross-presentation to CD8^+^ T-lymphocytes (24) but without affecting the levels of HLA class I expression at the cell membrane (5, 25). Therefore as control we tested the expression of HLA class I at the cell surface by flow cytometry. The results indicate no changes in HLA class I expression in any treated samples when compared to control, during Mtb mono-infection. However, during co-infection with HIV, we observed a significant increase in the expression of HLA class I in control cells, relatively to control cells mono-infected with Mtb. This result confirms the overall induced effect of cytosolic viral peptides in increasing the expression level of HLA class I molecules (**Figure 3A** lower panel). The results also indicate that the treatment with SQV and RTV induced no differences on the expression of the antigen presentation machinery.

Since BCG has been used as TB vaccine for more than one century, and since it has been losing the efficacy to protect from infection, we next tested the effect of PIs in improving the HLA class II expression at the cell surface, required to improve antigen presentation. As observed in **Figure 3C** for BCG infected cells we noticed a highly significant (*P* < 0.01) increase in antigen presentation levels.

To further evaluate the consequences of PIs treatment on antigen presentation we performed cocultures of treated and infected Mø with autologous CD4**^+^** T-lymphocytes obtained from the same healthy PPD^+^ donors and evaluated their ability to induce T-cell proliferation (**Figure 3D**). Following the same pattern of HLA surface expression, treatment with SQV in Mtb or BCG-infected cells induced a significant T-cell proliferation relatively to the control, after 5 days post-cocultures as evaluated by flow cytometry (**Figure 3D**). No changes were observed in cells treated with RTV.

We inferred that the induced T-cell proliferation would be concomitant with enhanced IFN-γ secretion and lately to indirect Mø activation and again the potentiation of the bactericidal effect. Therefore we performed quantification of IFN-γ secretion from non-infected cells and compared to infected ones treated or not with SQV. We observed in agreement with T-cell proliferation increased secretion of IFN-γ in cocultures supernatants of Mtb or BCG infection exacerbated in conditions treated with SQV **(** **Figure 3E** **)**; no significant alterations in IFN-γ secretion were detected in non-infected cocultures.

## Discussion

The purpose of our study was to decipher whether the first-generation HIV protease inhibitor, SQV, could be repurposed as a host-directed therapy for tuberculosis especially during co-infection with HIV. Tuberculosis, the so call white plague disease until the beginning of the twentieth-century, remains a leading cause of mortality worldwide due to an infectious agent. While in the last hundred years the vaccine BCG and the introduction of antibiotics helped to control the disease, since 1980 with the emergence of HIV this scenario has completely changed. HIV co-infection exacerbates Mtb infection helping reactivation from latency (2–4). Moreover due to increased drug resistance strains, co-infection impacts the transmission of MDR.

Accordingly, there is an urgent need to develop new medicines to control resistant bacteria and to redirect the immune responses of the host to effectively control the infection and the inflammatory responses. Within this context repurposing approved drugs will speed the process of improve therapy for the outcome of TB.

SQV was one of the first drugs developed to control HIV infection (26). It acts as an aspartic protease inhibitor interfering with HIV protease activity and therefore prevents the cleavage of Gag-Pol protein precursors. This inhibition ultimately blocks the infectivity of nascent virions and cell to cell spreading (10–12). It is likely that for other pathogens dependent on proteases for their life cycle, SQV and other PIs could be repurposed to control the respective infections. In fact they showed inhibitory effects against a wide spectrum of pathogens such as *Plasmodium falciparum* (27)*, Trypanosoma cruzei* (28), the fungi *Fonsecaea pedrosoi* (29) and SARS-CoV and avian influenza viruses (30).

It is expectable that HIV PI may also interfere with the host proteases. Among host proteases with relevance during immune responses to infections are cathepsins in the endocytic pathway and threonine proteases of the proteosome. Consistently, HIV PIs were shown to alter cathepsin activity of antigen presenting cells (13) and to interfere with proteosome peptide processing leading to accumulation of polyubiquitinated products (13, 21). Accordingly, HIV PIs designed to inhibit the HIV aspartyl protease were described to alter the activity of aspartyl proteases like cathepsin D and E, as well as cysteine proteases, such as cathepsin S (13).

Our previous results indicated the ability of Mtb to down-regulate the activity of cathepsins in order to successful survive in human Mø (14). Mtb infection leads to a strong inhibition of cathepsins B, S and L (14, 15) all of them involved in crucial activities during innate and adaptive immune responses. These results lead us to hypothesized that SQV, by inducing an increased activity of these cathepsins in non-infected immune cells, could be repurposed in the TB context to revert the blockade induced by the pathogen.

Here we demonstrated that SQV is able to increase omnicathepsins proteolytic activity during Mtb infection and during co-infection with HIV **(** **Figure 1A** **)**. In Mø, these endolysosomal enzymes are enrolled in pathogen killing as one of the first innate immune responses to infections. Likewise we observed a significantly intracellular killing of Mtb in human Mø treated with SQV **(** **Figure 2A** **)**. Since either the infection with these pathogens and the treatment of host cells with SQV are inducers of apoptosis (13, 21, 31, 32), we disregard this programmed cell death as inducer of pathogen killing by adjusting the experimental conditions **(** **Figure 1B** **)**. We may conclude that SQV induced pathogen killing was due to an increased activity of cathepsins along the endocytic pathway coincident with the same compartment of Mtb.

Cathepsin S contributes to the antigen presentation machinery by processing pathogen peptides and by generating of HLA-classII epitopes. Likewise, in infected cells treated with SQV a significant increase in HLA class II molecules were detected at the plasma membrane of infected cells leading to increased T-lymphocyte priming and proliferation **(** **Figures 3A, D** **)**. This was particularly relevant during BCG infection indicating that SQV improves the capabilities of presenting vaccine antigens **(** **Figures 3C, D** **)**. Since the population in Portugal has been vaccinated for decades with BCG until 2017, most of the population are PPD+. Thus we expect the blood from healthy donors to carry a significant population of memory/effector T cells that responded to the challenge of Mtb-infected macrophages (33, 34).

Here we found that SQV treatment induces higher levels of T-cell-secreted IFN-γ in a context that mimics bacteria replication during active TB **(** **Figure 3E** **).** This increased secretion of IFN-γ may have a dual effect: (1) activation of Mø to a more bactericidal state and, (2) indirectly contributing to a decreased IL-1β secretion. In fact it was previous demonstrated that pretreatment of *M. tuberculosis*–infected Mø with IFN-γ specifically inhibited the release of IL-1β suggesting that during TB IFN-γ may suppress lung immunopathology induced by dysregulation of IL-1β (35). These results suggest the cytokine environment might help achieve a better control of the immunopathology in the lungs, in accordance to published studies performed in murine models of Mtb mono-infection (35).

Moreover SQV has been referred to possess anti-inflammatory effects especially in the lungs (36). This was attributed to the suppression of TLR4 signaling pathways of high-mobility group box 1 (HMGB1). The beneficial effects were linked to decreased levels of circulating and lung tissue inflammatory cytokines, such as IL-6, IL-1β, TNF-*α*, and iNOS.

Cathepsins S and L have been demonstrated to regulate autophagy (37). Mtb and HIV are known to inhibit autophagy: upon infection of Mø in the lungs, inhibition of the autophagic pathway by the first invader will likely benefit the second or induce a similar behavior in neighboring cells (38). It could be that an SQV-induced increase of the proteolytic activities of cathepsins S and L would improve autophagy. This would in turn help infected cells to eliminate not only of the pathogens but also of cytosolic aggregates and inflammatory signaling molecules, contributing to decreased tissue inflammation (39).

Altogether our data and relevant literature indicates SQV as a potential host directed therapy for Tuberculosis.

## Data Availability Statement

The original contributions presented in the study are included in the article/**Supplementary Material**. Further inquiries can be directed to the corresponding author.

## Author Contributions

Conceptualization: EA, DP, JA-P. Methodology, acquisition and analysis: DP, EA. Investigation: DP, SV, MC, MM. Writing: EA. Supervision: EA. All authors contributed to the article and approved the submitted version.

## Funding

This study was supported by grants from National Foundation for Science, FCT Fundação para a Ciência e Tecnologia – Portugal, PTDC/SAU-INF/28182/2017 to EA.

## Conflict of Interest

The authors declare that the research was conducted in the absence of any commercial or financial relationships that could be construed as a potential conflict of interest.
