# Supplementary figures and images for: Repurposing Saquinavir for Host-Directed Therapy to Control Mycobacterium Tuberculosis Infection
Source: Front Immunol. 2021 Mar 26;12:647728. doi: 10.3389/fimmu.2021.647728 (PMC8032898; doi:10.3389/fimmu.2021.647728)

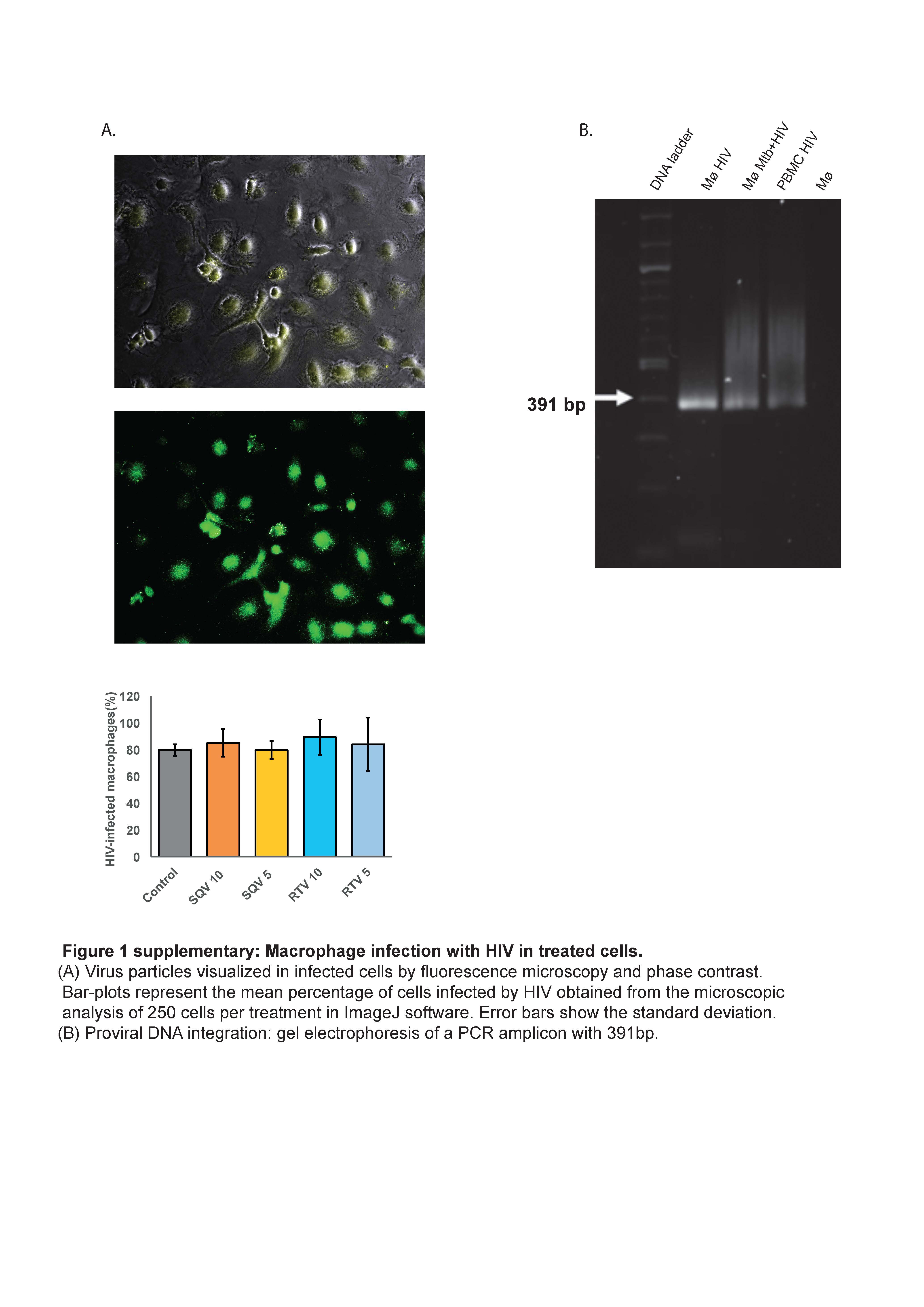

Supplement: Supplementary file 1 [file Image_1.jpeg]

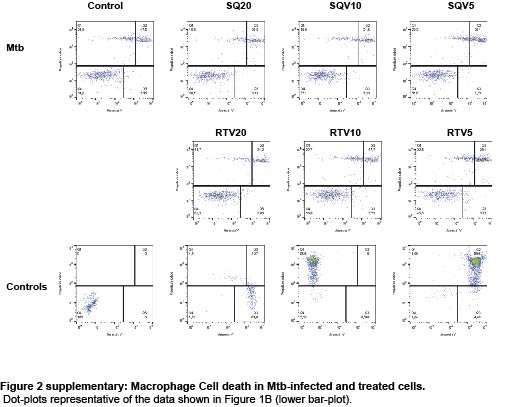

Supplement: Supplementary file 2 [file Image_2.jpeg]

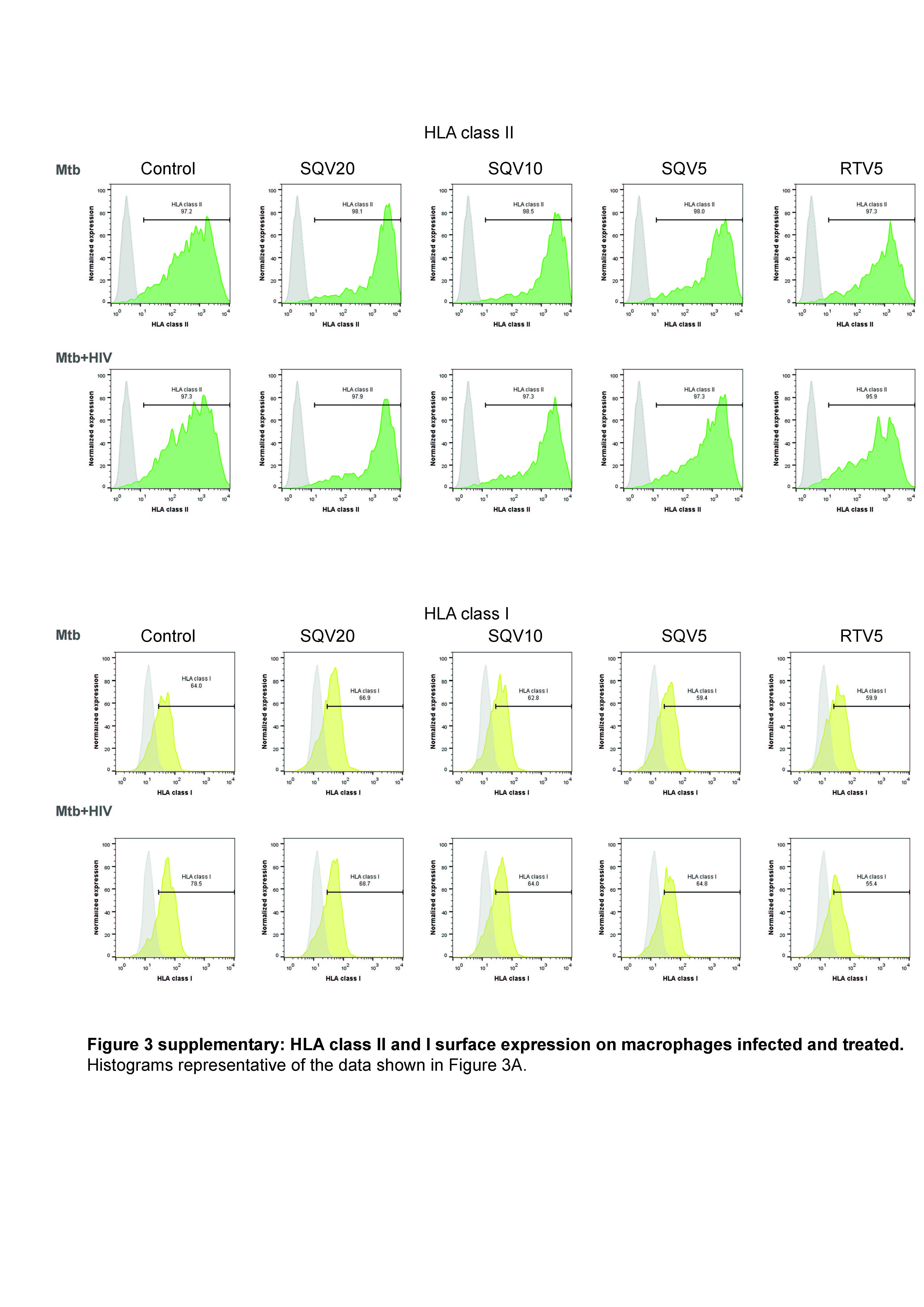

Supplement: Supplementary file 3 [file Image_3.jpeg]

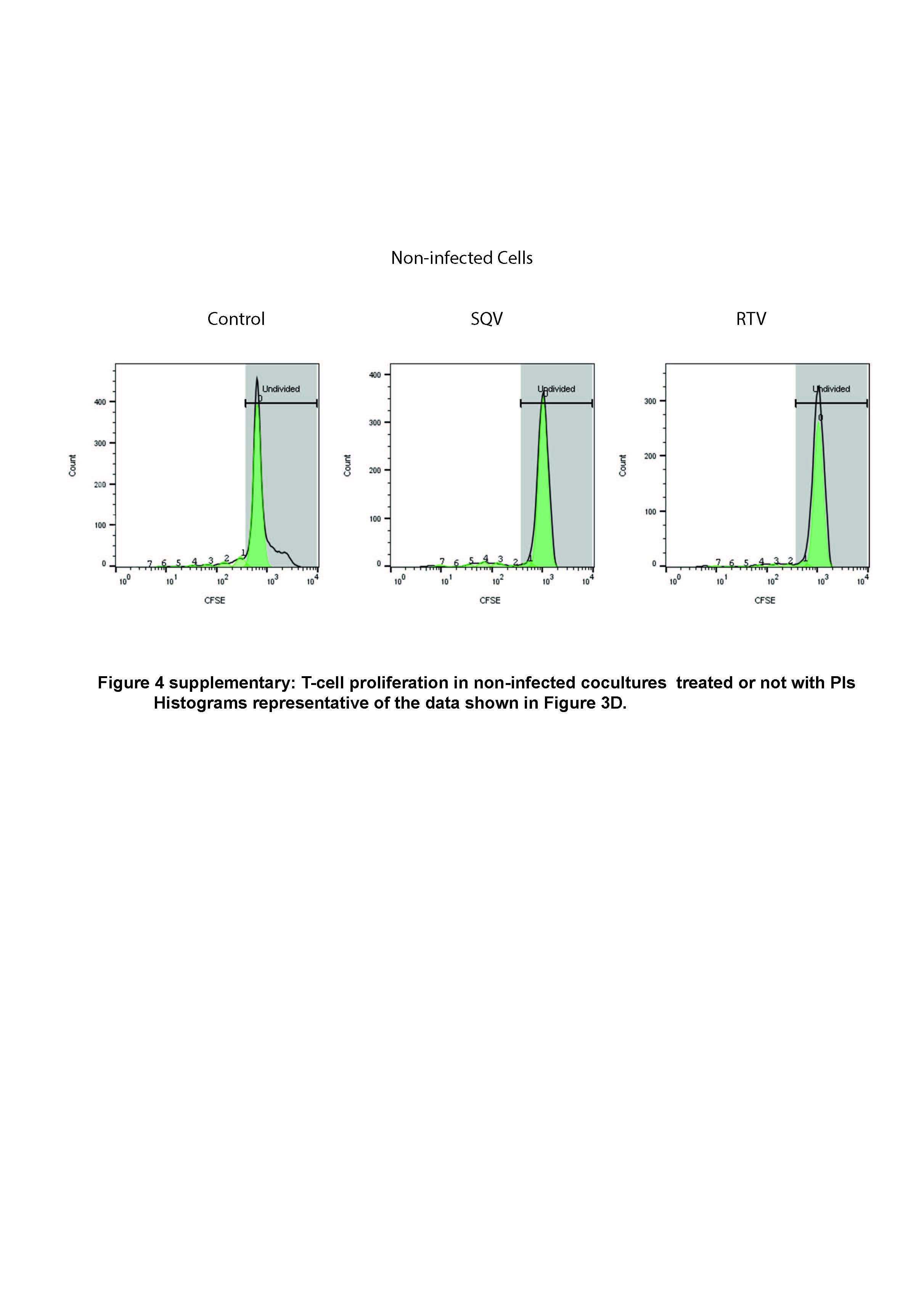

Supplement: Supplementary file 4 [file Image_4.jpeg]
